# Exploring the Utility of Web-Based Social Media Advertising to Recruit Adult Heavy-Drinking Smokers for Treatment

**DOI:** 10.2196/jmir.5360

**Published:** 2016-05-18

**Authors:** Krysten W Bold, Tess H Hanrahan, Stephanie S O'Malley, Lisa M Fucito

**Affiliations:** ^1^ Yale School of Medicine Department of Psychiatry New Haven, CT United States; ^2^ Yale Cancer Center New Haven, CT United States; ^3^ Smilow Cancer Hospital at Yale-New Haven New Haven, CT United States

**Keywords:** smoking, alcohol drinking, social media, research subject recruitment

## Abstract

**Background:**

Identifying novel ways to recruit smokers for treatment studies is important. In particular, certain subgroups of adult smokers, such as heavy-drinking smokers, are at increased risk for serious medical problems and are less likely to try quitting smoking, so drawing this hard-to-reach population into treatment is important for improving health outcomes.

**Objective:**

This study examined the utility of Facebook advertisements to recruit smokers and heavy-drinking smokers for treatment research and evaluated smoking and alcohol use and current treatment goals among those who responded to the Web-based survey.

**Methods:**

Using Facebook’s advertising program, 3 separate advertisements ran for 2 months targeting smokers who were thinking about quitting. Advertisements were shown to adult (at least 18 years of age), English-speaking Facebook users in the greater New Haven, Connecticut, area. Participants were invited to complete a Web-based survey to determine initial eligibility for a smoking cessation research study.

**Results:**

Advertisements generated 1781 clicks and 272 valid, completed surveys in 2 months, with one advertisement generating the most interest. Facebook advertising was highly cost-effective, averaging $0.27 per click, $1.76 per completed survey, and $4.37 per participant meeting initial screening eligibility. On average, those who completed the Web-based survey were 36.8 (SD 10.4) years old, and 65.8% (179/272) were female. Advertisements were successful in reaching smokers; all respondents reported daily smoking (mean 16.2 [SD 7.0] cigarettes per day). The majority of smokers (254/272, 93.4%) were interested in changing their smoking behavior immediately. Many smokers (161/272, 59.2%) also reported heavy alcohol consumption at least once a month. Among smokers interested in reducing their alcohol use, more were heavy drinkers (45/56, 80.4%) compared to non-heavy drinkers (11/56, 19.6%; χ^2^[_1,N=272_]=13.0, *P*<.001). Of those who met initial screening eligibility from the Web-based survey, 12.7% (14/110) attended an in-person follow-up appointment.

**Conclusions:**

Social media advertisements designed to target smokers were cost-effective and successful for reaching adult smokers interested in treatment. Additionally, recruiting for smokers reached those who also drink alcohol heavily, many of whom were interested in changing this behavior as well. However, additional social media strategies may be needed to engage individuals into treatment after completion of Web-based screening surveys.

## Introduction

Identifying novel ways to recruit smokers for treatment is important. Tobacco use remains a large public health problem [1,2], and most smokers who try to quit each year do so without any form of treatment (68.3%) [3] and rarely achieve lasting abstinence [3]. Certain subgroups of adult smokers, such as heavy-drinking smokers, may be especially at risk given evidence that they are less likely to try quitting smoking [4-7] and have greater odds of serious medical problems, including cancers, liver cirrhosis, and pancreatitis [8-11]. Thus, drawing smokers and heavy-drinking smokers into treatment is important for improving health outcomes.

Research suggests there is a large subgroup of smokers who also drink heavily. In national samples, approximately 6.2 million US adults have co-occurring nicotine dependence and an alcohol use disorder [12]. Additionally, rates of binge (ie, ≥5 drinks on the same occasion) and heavy alcohol use (ie, binge use ≥5 times in the past month) are more than twice as high in smokers compared with non-smokers (binge: 42.9% smokers, 17.5% nonsmokers; heavy drinking: 15.7% smokers, 3.8% nonsmokers) [13].

Although most heavy drinkers do not seek treatment [14], smoking status may be a way of identifying heavy drinkers [15] and engaging them in treatment. Recently, Internet-based recruitment strategies have been evaluated as alternative methods for reaching and engaging individuals into treatment and clinical research. Social media advertising, such as through Facebook, was more cost-effective for reaching young adult smokers in a national Web-based survey study, totaling $4.28 per survey, and produced more valid results than other Web-based advertisements (eg, Craigslist) or recruitment methods through survey companies [16]. Another study indicated that Web-based recruitment captured a greater proportion of people with substance use and mental health issues compared with alternative recruitment methods (ie, flyers, university courses) [17]. Others have successfully used social media advertising to reach targeted audiences for substance use research, including difficult-to-recruit populations such as young adults [18], veterans [19], immigrants [20], and Latino smokers [21]. These studies suggest Web-based advertising may be a useful tool to consider for recruiting heavy-drinking smokers as well.

Given the widespread use of social media by the general adult population [22], and research highlighting the usefulness of Web-based recruitment strategies, we sought to examine the utility of Facebook advertising to recruit adult smokers from the community for treatment. The primary aims of the study were to (1) evaluate the effectiveness of Facebook advertising to reach adult smokers and a subpopulation of heavy-drinking smokers and (2) characterize smoking and alcohol use and current treatment goals among responders. Identifying who responds to smoking advertisements on social media may suggest new ideas for recruitment or innovative Web-based interventions to address substance use.

## Methods

### Participants

The target population was adult smokers living in the greater New Haven, Connecticut, area who were interested in participating in smoking cessation treatment research. Individuals had to be literate in English to complete the Web-based survey.

### Facebook Recruitment

**Figure 1:**
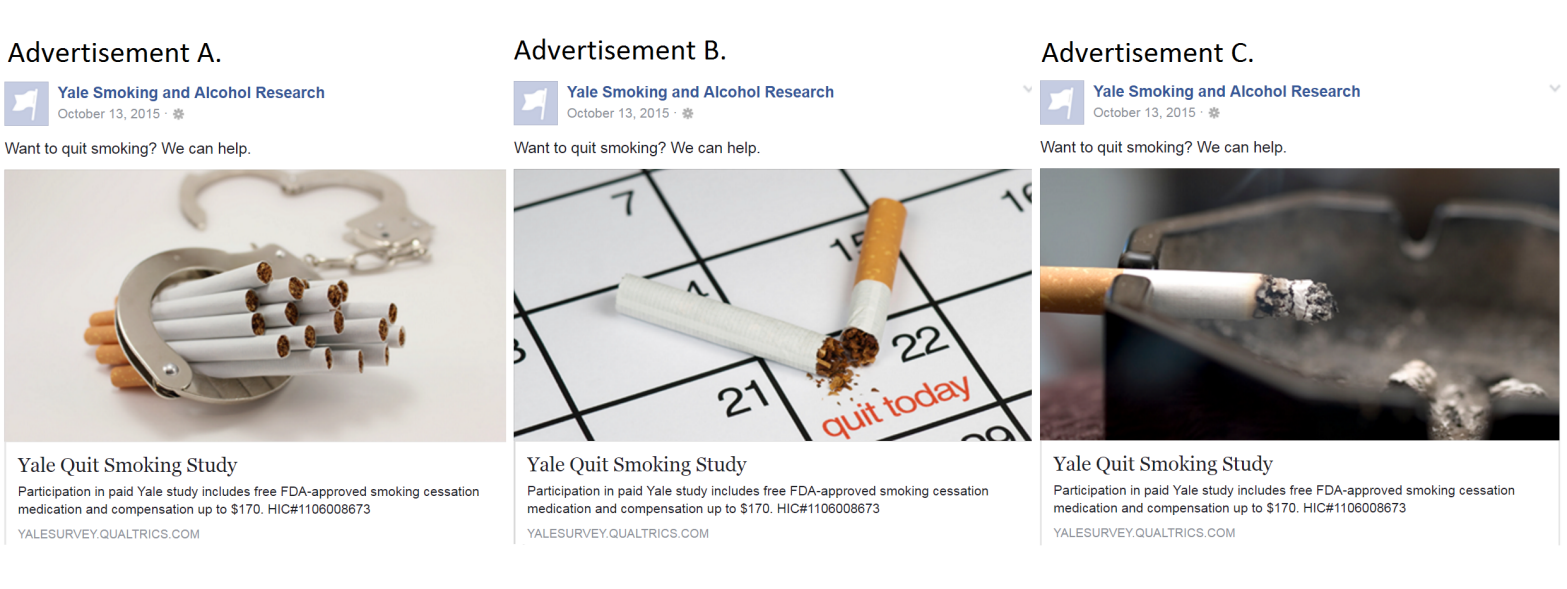
Facebook advertisements designed to target smokers interested in quitting.

We utilized Facebook’s advertising program for 2 months from April 13, 2015 through June 16, 2015. We created 3 advertisements that appeared on the Facebook pages of our target audience (at least 18 years old) within a geographic radius of 30-40 miles from New Haven, Connecticut, given our interest in recruiting individuals able to participate in an in-person treatment research study. All 3 advertisements targeted smokers interested in treatment by presenting images of cigarettes with the phrase “Want to quit smoking? We can help” (Figure 1). Facebook approved all advertisements. The presentation of advertisements to users was not randomized; all 3 advertisements were uploaded simultaneously to the Facebook platform with identical settings. The Facebook advertisement delivery system was set to optimize advertisement presentation over time. This system rotates through the advertisement presentation to display all 3 advertisements in the set, and when it is determined that an advertisement is performing better than others (ie, greater number of clicks), this advertisement is selected by the program to be displayed more often.

We selected days when we wanted the advertisements to run; the advertisements ran for 14 days in total over 2 months. We specified a spending limit for the entire advertising campaign (3 advertisements) of $25 per day. On days when advertisements were running, Facebook set pricing for the campaign (ie, a “bid”) that was used to calculate a competitive value (ie, sum of the bid plus the advertisement’s intrinsic quality, measured by past performance). Bids are entered into an auction where all advertisers compete for their advertisement to be shown to their target audience. Facebook monitors competing bids from other advertisers and optimized our advertisement pricing based on our spending limit to enhance the likelihood that the advertisement would win an auction and be visible to Facebook users [23]. Facebook monitored the number of impressions (ie, each time an advertisement is shown to a user), total reach (ie, number of people seeing the advertisements), clicks on the advertisements, and total cost for all advertisements. We used this data to evaluate the success of each advertisement and cost-effectiveness in obtaining survey responses. In accordance with Facebook’s data use agreement, we present aggregated anonymous results.

### Study Procedures

The Yale Institutional Review Board approved all study procedures. After clicking on the advertisement, participants were brought to a website that described the purpose of the survey for determining preliminary eligibility for a treatment research study. Participants were informed that their responses were confidential and were told they could skip questions they did not wish to answer although this may affect their eligibility. Participants were given contact information for research staff if they had questions or preferred to complete the screening by phone. Participants provided informed consent by selecting “yes,” indicating they understood this information, were at least 18 years old, and wished to complete the Web-based survey. Participants were not offered any incentives for completing the survey, but the advertisement indicated paid participation in the research study (up to $170) and free FDA-approved smoking cessation treatment if eligible. Survey participants entered contact information (email addresses and/or phone number) to facilitate scheduling an intake for the treatment research study. Responses were screened for duplicate entries and were excluded from the current analysis if the same email address or phone number was listed across multiple surveys.

### Assessments

The Web-based survey contained 18 questions and took 5-10 minutes to complete. Multiple-choice responses provided an option for participants to select “No” or “I choose not to answer.”

#### Demographics

To ensure that Web-based surveys were brief, data were only collected about participant age and sex, which were used to assess initial eligibility for the main treatment research study (eg, females who were currently pregnant or nursing were excluded).

#### Smoking History

Participants reported the number of cigarettes they currently smoke and their interest in reducing their cigarette use rated “immediately,” “at a later date,” “I am not interested in reducing my cigarette use,” or “I choose not to answer.”

#### Alcohol Use History

Frequency of heavy alcohol use was assessed by asking participants to select how frequently they consumed 4 or more (if female) or 5 or more (if male) standard drinks using National Institute on Alcohol Abuse and Alcoholism (NIAAA) guidelines for heavy drinking [24]. Categorical response options included “every day,” “5 to 6 times a week,” “3 to 4 times a week,” “1 to 2 times a week,” “2 to 3 times a month,” “once a month,” and “never.” Participants were provided information about standard drink equivalents (ie, 12-ounce bottle of beer, 5-ounce glass of wine, one shot of hard liquor by itself or in a mixed drink). Desire to reduce alcohol use was rated “yes,” “maybe at a later date,” or “no.” Alcohol withdrawal symptoms were assessed by asking participants if they experienced 10 specific symptoms when they cut down or stopped drinking (eg, sweating, vomiting, seizure, hallucinations) rated “yes,” “no,” “I choose not to answer,” or “N/A” if participants had never cut down or stopped drinking.

#### Other Eligibility Screening Questions

Screening questions regarding whether participants would be available and eligible for the in-person treatment program included “are you currently taking part in any other research study,” “do you have a permanent address and phone number (yes or no),” and “are you planning on moving out of the greater New Haven area in the next 6 months?” Additionally, participants were asked if they were currently pregnant or nursing, being treated for any medical problems, currently taking any prescription medications, and had a history of treatment for psychiatric problems. They were prompted to provide free-response text to specify the condition or medication.

### Initial Screening Eligibility

Completed surveys were evaluated by research staff to determine initial eligibility for participation in a treatment research study. Eligibility criteria for being invited for an in-person intake evaluation included being at least 18 years old, currently smoking cigarettes, reporting at least 1 heavy drinking day in the past month, no history of severe alcohol withdrawal (eg, hallucinations), not currently participating in another research study, reporting having a permanent address and phone number, not planning to move in the next 6 months, not currently pregnant or nursing, not currently using medication to treat alcohol or tobacco use (eg, naltrexone, varenicline, bupropion, nicotine replacement), and no history of serious psychiatric illness (eg, schizophrenia, bipolar disorder).

## Results

### Recruitment Results

**Figure 2:**
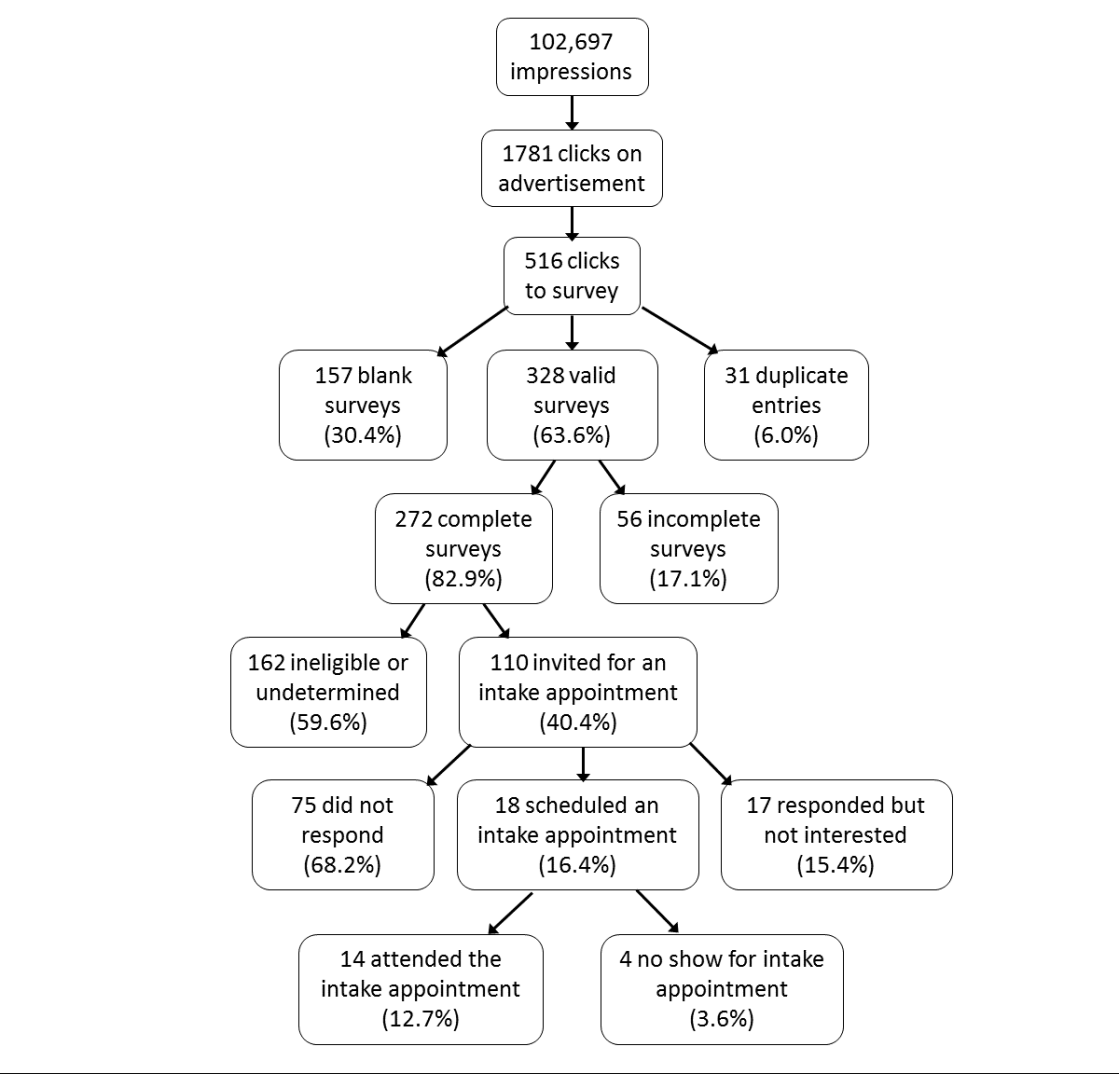
Flow diagram showing response rates to Facebook advertisements.

Recruitment results are depicted in Figure 2. In total, 3 Facebook advertisements generated 102,697 impressions (ie, number of times the advertisement was displayed), 1781 clicks on the advertisement, and 516 surveys in 2 months, at an overall cost of $480.89. Facebook estimated we had a daily reach (ie, number of people who saw the advertisement) of 3400-9100 out of 890,000 (the total number of people in our selected audience who were active on Facebook each day). The majority of people viewed and clicked on our advertisements from mobile devices (1662/1781, 93%, vs 113/1781, 6% on tablets and 6/1781, <1% on desktops). Of the 3 advertisements, advertisement A (Figure 1) generated the most interest and clicks to the survey page (1393/1781, 78.2% of clicks). A total of 328 valid Web-based surveys were analyzed after removing blank (n=157) and duplicate (n=31) entries. Of the 328 surveys, 55 (16.8%) answered only initial questions related to demographics and interest in changing smoking or drinking behavior. Completed surveys were reviewed, resulting in 110 participants who met preliminary screening criteria. Research staff contacted all 110 participants to let them know more about the study and invite them to complete an intake and further evaluation for a treatment research study. Of those, 18 were interested in the treatment study and scheduled an intake appointment. Most participants who scheduled an intake (14/18) attended this appointment. Overall, Facebook advertising was highly cost-effective, averaging $0.27 per click, $1.76 per completed survey, $4.37 per participant eligible for an intake, and $34.35 per participant who completed the in-person intake.

### Participant Characteristics

Descriptive statistics were used to characterize participant demographics and responses to survey items. On average, survey responders were 37.4 (SD 10.8) years old, and 63.4% (208/328) were female. Independent sample *t* tests and chi-square analyses were used to assess differences in demographic characteristics by survey completer status (Table 1). Age (*t*_325_=−1.86, *P*=.06), desire to reduce cigarette use (χ^2^[_2,N=328_]=0.6, *P*=.73), and desire to reduce alcohol use (χ^2^[_2,N=328_]=2.3, *P*=.32) did not differ significantly by completion status. Significantly more females completed the survey (179/272, 65.8%) than males (93/272, 34.2%; χ^2^[_1,N=328_]=3.9, *P*=.04).

**Table 1:** Demographic characteristics by survey completion status.

Demographic characteristics		Incomplete survey (N=56)	Complete survey (N=272)
Age in years, mean (SD)		39.8 (12.2)	36.8 (10.4)
Sex, female, n (%)		29 (51.8)	179 (65.8)
**Desire to reduce cigarette use, n (%)**			
	Immediately	53 (94.6)	254 (93.4)
	At a later date	3 (5.4)	15 (5.5)
	No response	0 (0.0)	3 (1.1)
**Desire to reduce alcohol use, n (%)**			
	Yes	15 (26.8)	56 (20.6)
	Maybe at a later date	9 (16.1)	32 (11.8)
	No	32 (57.1)	184 (67.6)

### Smoking and Alcohol Use

**Figure 3:**
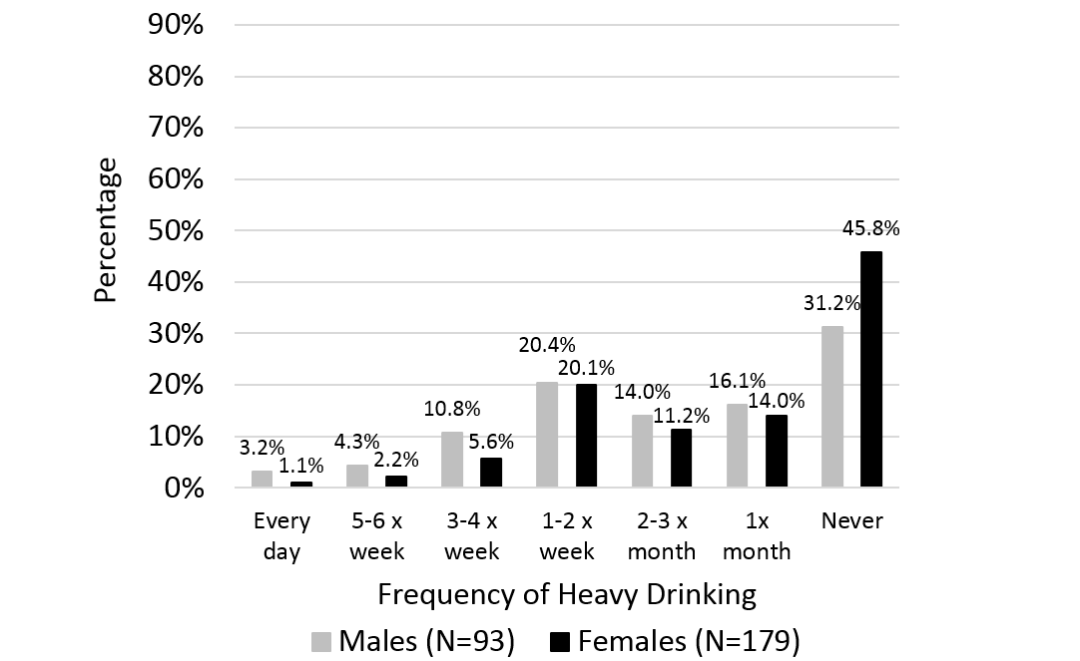
Frequency of heavy alcohol use by sex.

The remaining analyses characterized responses from the 272 completed surveys. The advertisement was successful in reaching smokers interested in treatment. All survey respondents (n=272) reported daily smoking and the majority stated an interest in immediately reducing their cigarette use. Subjects reported smoking 16.2 cigarettes (SD 7.0) per day on average. Additionally, many of the smokers who responded also regularly used alcohol. More than 59% (161/272) reported heavy alcohol consumption at least once a month, measured according to NIAAA guidelines as ≥ 5 standard drinks/men, ≥ 4 standard drinks/women [24]. Figure 3 shows the frequency of heavy drinking reported by men and women in the sample. More than 19% (54/272) of respondents indicated they that experienced at least one alcohol withdrawal symptom in the past.

Although most respondents did not wish to reduce their alcohol use at the time of completing the survey, almost one-third were interested in making changes now or in the future. Chi-square analyses were used to examine whether treatment interest differed between those who reported heavy drinking at least once a month and those who reported never experiencing heavy drinking. The majority of smokers also interested in reducing their alcohol use now were heavy drinkers (45/56, 80.4%) compared to non-heavy drinkers (11/56, 19.6%; χ^2^[_1,N=272_]=13.0, *P*<.001).

### Medical and Psychiatric History

Participants responded to questions about current medical and psychiatric problems to assess initial eligibility. Overall, 1.5% (4/272) were currently pregnant or nursing, 22.1% (60/272) reported currently being treated for a medical problem, 17.3% (47/272) reported a history of treatment for psychiatric problems, 31.6% (86/272) reported currently taking medications or prescription drugs, and less than 5% (13/272) selected that they preferred not to answer these questions.

## Discussion

This study evaluated the utility of Facebook advertising to recruit smokers and heavy-drinking smokers for treatment. Using 3 advertisements over 2 months, we were able to target adults interested in smoking cessation treatment, many of whom also drank heavily and were interested in changing their alcohol use as well. The majority of clicks on the survey from our advertisements resulted in valid, completed survey responses. Furthermore, the obtained information allowed for a cost-effective way of conducting initial eligibility screening, although additional efforts may be needed to engage individuals into treatment after completion of Web-based screening surveys.

Overall, Facebook was effective for reaching adult smokers and heavy-drinking smokers. However, only 12.7% of those who were initially eligible based on survey responses attended an in-person intake meeting for treatment research. The average cost per valid completed survey was comparable to costs from social media advertising reported by Ramo and colleagues [16]. Although the cost per participant completing the intake appointment was higher ($34), this was comparable or cheaper than other investigations using Web-based social media to recruit for smoking studies ($30-$170) [25-28], and was less expensive than traditional recruitment costs (eg, direct mailing or print journalism) reported in other studies ($50-$600) [26,29,30]. Only a small proportion started but did not complete the survey. We successfully obtained responses about tobacco and alcohol use for the majority of respondents who initiated the survey, suggesting Web-based surveys may be especially useful for quickly obtaining a range of information from target audiences, including information on potentially stigmatized behavior such as substance use. Additionally, elements of online communication such as convenience, familiarity, or anonymity may contribute to individuals’ greater willingness to complete Web-based surveys compared with face-to-face meetings. Although this may reduce the rate of in-person follow-up from Web-based advertising and potentially limit the utility for recruiting smokers into traditional treatment in practice, it is also possible that Web-based advertising reaches individuals who would not otherwise present for treatment. Identifying more effective ways to encourage in-person follow-up or capitalize on online convenience will be important for treatment dissemination.

In particular, reaching heavy-drinking smokers through smoking cessation treatment may provide unique opportunities for motivational enhancement interventions for those who are not currently interested in treatment for their alcohol use. Although most survey respondents were interested in changing their smoking behavior, fewer were interested in changing their alcohol use. However, our results suggest there is a subset of smokers who are interested in changing both tobacco and alcohol use behaviors at the same time. These findings are consistent with prior work [31,32] and support the idea of integrating smoking cessation services into treatment for co-occurring substance use [33-36].

This study has several limitations that should be considered. First, we are unable to determine the representativeness of the sample recruited through Facebook. Data generated by Facebook do not allow us to assess how our sample of respondents compares with the general population of Facebook users meeting our target criteria. Additionally, our sample is from a limited geographic region given our interest in using Web-based recruitment to generate participants for an in-person treatment study. We only collected demographic information related to sex and age, thus we are limited in our ability to determine how representative this sample was of our geographic region or how these results may compare with other regions. However, the average age and cigarettes per day of our sample were similar to other studies using Internet-based recruitment for adult smokers [25,37], supporting the generalizability of these findings. Furthermore, our 3 advertisements were viewed an unequal number of times as designed by the Facebook algorithm (ie, more popular advertisements were shown more often), and without systematically varying the advertisement content and presentation of images, it is difficult to determine which elements of advertisement A were most appealing. It is possible that something about the advertisement itself resonated more with the audience, although additional work is needed to determine the most effective social media advertising image. Lastly, we are limited by the self-report nature of the assessments, and survey results may be influenced by reporting bias. We are unable to determine the reason for incomplete survey responses. Researchers should carefully consider participant factors or other technological factors that may affect Web-based survey completion.

Despite these limitations, our results suggest Facebook advertisements were successful in reaching adult smokers interested in treatment research. Additionally, general recruitment for smokers reached those who also drink alcohol heavily, which may provide an opportunity to engage heavy drinkers into treatment. Given the widespread use of social media and the ease of connecting to users on the go through mobile devices, Web-based advertising for survey or intervention research may be an important strategy to engage at-risk and hard-to-reach populations.

## Abbreviations

NIAAA: National Institute on Alcohol Abuse and Alcoholism
FDA: Food and Drug Administration
